# Efficacy of Mindfulness-Based Cognitive Therapy on Quality of Life of Mothers of Children with Cerebral Palsy

**Published:** 2015-04

**Authors:** Zahra Sedaghati Barog, Seyyed Jalal Younesi, Amir Hosein Sedaghati, Zohre Sedaghati

**Affiliations:** 1 Department of Counseling, University of Social Welfare and Rehabilitation Sciences, Tehran, Iran; 2 Department of Counseling, University of Social Welfare and Rehabilitation Sciences, Tehran, Iran; 3 Kalamstan language foundation manager (CEO), unit 4-second floor-number 735-Shahid Ghandi Square-North Sohrevardi- Seyed Khandan Bridge-Tehran-Iran.; 4 rights expert,assistance.

**Keywords:** *Cerebral Palsy*, *Cognitive Therapy*, *Mindfulness*, *Quality of life*.

## Abstract

**Objective**: The findings demonstrated that parents of children with cerebral palsy experience elevated levels of distress, depression, anxiety, posttraumatic stress symptom and subjective symptom of stress and low quality of life. Effective interventions targeting relapse have the potential to dramatically reduce the point prevalence of this condition.

Many studies have shown that Mindfulness Based Cognitive Therapy (MBCT) is an intervention that has shown efficacy in improving quality of life. In this study, the effect of Mindfulness –Based Cognitive Therapy (MBCT) on increasing quality of life in mothers of children with cerebral palsy has been examined.

**Method:** Three mothers of CP children with low scores on quality of life in WHOQOL-BREF inventory participated in this single- case study.

**Results**: Findings revealed that the MBCT program elevated quality of life of the participants. The improvement quotient for quality of life of each participant was good .

**Conclusion:** The results have implication for efficacy of mindfulness for improvement of psychosocial life of families of children with cerebral palsy.

Quality of life (QOL) describes an individual’s perception of his or her position in a context in relation to concerns and goals (1). A child with cerebral palsy (CP) may cause physical and psychological stress in the family (2-4) because this disorder is a collection of motor disorders resulting from a damage to the child’s brain which affects the motor system and causes poor coordination, poor balance, abnormal movement patterns or combination of these characteristics, delay speaking or incomprehensible speech (5, 6). Therefore the psychological and physical health of caregivers is strongly influenced by the child’s behavior and the caregiving demands (7, 8).

The most affected person in the family is usually the mother in such a situation. Mothers of children with disabilities often experience greater stress and emotional demands than do other mothers (9). The difficult and constant struggle to improve the child's health and development is accompanied by doubt, guilt and shame, which contributes to the deterioration of the quality of life of these mothers. Experiencing severe anxiety (e.g., before making a crucial decision) oftentimes leads to feelings of helplessness and lack of control, and this in turn may contribute to the feelings of maternal incompetence. Fatigue and frequent loneliness reduce resistance to stress and disturb the normal regulation of emotions (10).Dispositional mindfulness is associated with greater life-satisfaction and self-esteem (11). In previous studies and this study (12, 13), it was found that participants benefited from mindfulness-based cognitive therapy (MBCT) in order to fight depression and anxiety, which are normally induced by a real stressful setting. In this study, we aimed to examine whether MBCT is effective in enhancing quality of life of mothers of children with cerebral palsy.

People undergoing such a training program learn to understand thoughts, feelings and bodily sensations as passing events in the mind rather than self-evident truths or aspects of the self. By this approach, the skills learned from MBCT help people recognize and disengage themselves from habitual dysfunctional cognitive routines, which in turn protect them against future risks of experiencing anxiety and depression. There are also studies showing that MBCT reduces excessive worry or anxiety symptoms (14), relieves insomnia symptoms by reducing worry associated with sleep problems in patients with anxiety disorder (15) and improves quality of life in physical and psychological domains (16).

 Therefore, in this study, it was hypothesized that providing MBCT training to the participants may increase their quality of life significantly.

## Material and Methods

*Participants*

The participants of this study were three mothers who were selected from those mothers being referred to the University of Social Welfare and Rehabilitation Centers for the rehabilitation of their children. The facilitator screened the interested mothers based on the inclusion and exclusion criteria. Inclusion criteria were obtaining a score of 60 or less in WHOQOL-BREF, and being medically stable. Patients with physical illness, substance abuse and /or dependence and psychosis and disability were excluded from the study due to low concentration and orientation. All children were boys with cerebral palsy spastic type [Ages 4-6]. All children lived with their parents.

*Procedure*

This article was part of a larger research work of the student's thesis. The method used in this study was the single- case experimental design. In analyzing the data in the single case study, the dependent variable for the possible changes resulting from the independent variable can be read in two ways. The first criterion is to draw the graphs of the subjects’ functions at the baseline and the intervention phase, and then compare them; the second criterion is to consider the slopes in each of the two –step graph-line during the intervention (17). Thus, any trends or slopes in each stage are examined. In this study, the improvement quotient was used to show the treatment effect clearly. We subtracted the pretest scores from the post-test score and then divided the attained number by the pretest score. 

The baseline included three measures of maternal quality of life using the WHOQOL-BREF Inventory before the intervention. To monitor the changes, the measurements were also performed at the end of the second, forth, sixth and eighth sessions. The participants individually attended the treatment sessions which lasted one hour for eight consecutive weeks.

*Intervention Program (* 18 *,* 19 *)*

Mindfulness based Cognitive Therapy (MBCT) combines training in meditation and psycho education on cognitive processing to teach the followings: 

1. Attentional control—how to stay in present-moment sensory awareness without being distracted by strong emotions or thoughts (and to bring one’s focus back to a central object over and over again in order to tame the highly distractible mind)

2. How to disengage from judgmental, evaluative language based processing (doing) and move to an experiential awareness of the present moment (being)

3.Specifically how to apply the above skills to notice and disengage from negative thoughts and emotions and physical sensations 

Mindfulness based intervention consists of the development of a particular kind of attention, characterized by a nonjudgmental awareness, openness, curiosity and acceptance of internal and external present experiences, which allows the practitioners to act more reflectively rather than impulsively. 

In this study, the intervention was provided in eight sessions.

In the first session, goals and techniques included building a rapport with the client, obtaining information from the client, providing psycho education on mindfulness, CBT, depression, stress, anxiety, identifying automatics thoughts and leading the client through a guided mindfulness meditation.

In the second session, the goals and techniques included ‘Automatic pilot’ (acting without conscious awareness), having a childlike curiosity and mindful eating body scan (intentionally bringing awareness to bodily sensations).

In the third session, the goals and techniques included dealing with barriers (awareness of how the chatter of the mind influences feelings and behaviors), and being compassionate with oneself and short breathing meditation. 

The goals and techniques in the fourth session included helping the client recognize that most of her thoughts are not facts, and teaching her to use the thought record, and provide training on cognitive distortion.

The goals and techniques in the fifth session included teaching the client the ability to stay in the present time with awareness of attachment and aversion; then, diaphragmatic breathing and sleep hygiene were explained; next, the client was taught a brief body scans exercise to reduce muscle tension.

In the sixth session, the goals and techniques included the acceptance of thoughts and emotions as fleeting events; next, introducing mindful daily activity, teaching mindful eating and mindful labeling on thoughts, feelings and behaviors.

In the seventh session, the goals and techniques included familiarizing the clients with the symptoms of depression, stress and rumination thinking. Also, we trained them to accept their rumination thinking without judgment and to use the diffusion technique to reduce it.

In the eighth session, the goals and techniques included reviewing the insights and techniques found most useful by the client, identifying the obstacles to practice mindfulness, and providing a checklist of techniques included in the program.

*Instrument*

*WHOQOL-BREF*

To evaluate quality of life, we used the 26-item, self-report, short version of the World Health Organization Quality of Life instrument(20).This instrument provided us with a subjective quality of life assessment in four domains of physical (e.g., “How satisfied are you with your sleep?”), psychological (e.g., “How much do you enjoy life?”), social (e.g., “How satisfied are you with your personal relationships?”), and environment (e.g., “How satisfied are you with your access to health services?”). Data were reported on the total scores of the first three domains, as they were more relevant to the aims of this study. WHO (20) asserts that WHOQOL-BREF can be regarded and used as a cross cultural measure. The Farsi version of WHOQOL BREF was employed in the present study. One study (21) which was carried out in an Iranian population showed a good construct validity and internal consistency for the Farsi version of WHOQOL-BREF.

## Results

*Participant A*

Diagram1 shows that the participant’s quality of life level is was 55-56 in WHOQOL-BREF, approximately at the baseline level statement., which is considered a These scores are weak rates of quality of life in WHOQOL-BREF. She obtained a score of 60 in WHOQOL-BREF at the end of session 2; , and this increasing increase continued until the end of the intervention.as in In the last session, her score in the post-test measurement was 96 in WHOQOL-BREF. , which That indicates improvement in quality of life. Her Percent of recovery was %73.50% for quality of life.

*Participant B*

Diagram 2 shows demonstrates that the participant's quality of life levels were 48-50 in WHOQOL-BREF, approximately at the baseline statement level. These scores are weak ranges of quality of life in WHOQOL-BREF. She obtained a score of 52 in WHOQOL-BREF at the end of session 2. Her score in the last post-test measurement were was 96 in WHOQOL-BREF that indicates an increasing increase in quality of life. Her improvement quotient was %97.25% for quality of life.

*Participant C*

Diagram 3 shows displays that the participant's quality of life levels are were 57-59 in WHOQOL-BREF, approximately at the baseline statement level. These scores are weak rates of quality of life in WHOQOL-BREF. She got the score of 64 in WHOQOL-BREF at the end of session 2. 

Her score in the last post-test measurement were was 93 in WHOQOL-BREF that shows an increasing increase in quality of life. Her improvement quotient was %59.44% for quality of life.All participants showed scores of 48-59 at the baseline and this statement that indicated a low (-60) quality of life in WHOQOL-BREF. Visual the visual observation of diagrams demonstrates the increase of the scores. Post The post- test score of the three participants (A, B and C) is a moderate score of quality of life in WHOQOL-BREF.

Differences The differences between the base line and post-test scores demonstrated that subjects the participants showed positive improvement on WHOQOL-BREF scales. The percentages of recovery on quality of life were as follows: participant A: %73.50%; participant B: %97.25%; and participant C: %59.44%.

MBCT seems to have produced incremental benefits in quality of life. Thus far, A number of studies showed that mindfulness trainings enhance psychological and health-related quality of life in heterogeneous patients population (22).Our findings along with numerous other studies clearly indicate that the magnitude of the impact that MBCT can have on some individuals is substantial.

These results are consistent with recent studies investigating the relationship between mindfulness and predictors of quality of life. (22, 23).

The sub - Domains of quality of life 

The Results of WHOQOL-BREF shows that; MBCT has led to improved physical and mental health of the participants. In fact, MBCT seems to have produced incremental benefits in quality of life.(24-26) of the participants (24-26).There are also studies showing that MBCT reduces excessive worry or anxiety symptoms, relieves insomnia symptoms by reducing worry associated with sleep problems in patients with anxiety disorder(27). and Also, a number of studies have showing shown that mindfulness trainings enhance psychological and health-related quality of life in heterogeneous patients population(28) and reduces physical and mental health problems (19, 22, 29). In this study also, MBCT reduces physical and mental problems in the participants, but and it we also found also noted that mothers in this study said that; believed that this intervention could not to fill the vacancies requiring the participants to provide express their economic issues problems and receive support from the government or from their extended family members and support systems..

**Table 1:** Demographics Information of Mothers with Cerebral Palsy Children

	**Participant A**	**Participant B**	**Participant C**
**Age of child(years)**	6	4	4
**Gender**	Boy	Boy	Boy
**Type of cerebral palsy**	Spastic (Hemiplegia)	Spastic (Diplegia)	Spastic (Hemiplegia)
**Age of mother**	35	41	39
**Occupation of mother**	housewife	Typist at home	Housewife
**Number of children**	2	1	2

**Figure 1:**
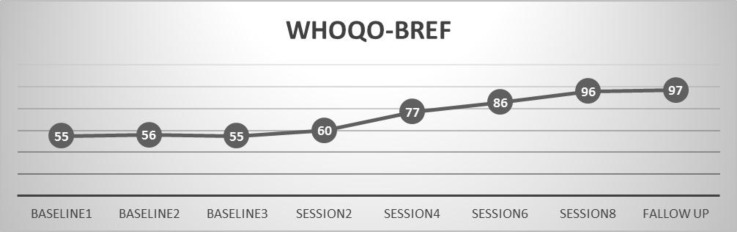
Quality of life in the three sessions before the intervention and eight session intervention for A mother with cerebral palsy child Based on WHOQOL-BREF, differences in 2, 4, 6, 8 sessions and follow up session were significant.

**Figure 2:**
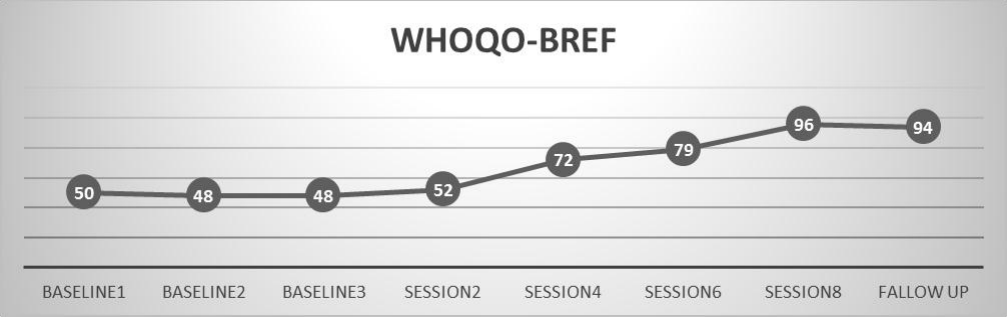
Quality of life in the three sessions before the intervention and eight session intervention for B mother with cerebral palsy child Based on WHOQOL-BREF, differences in 2, 4, 6, 8 sessions and follow up session were significant.

**Figure 3:**
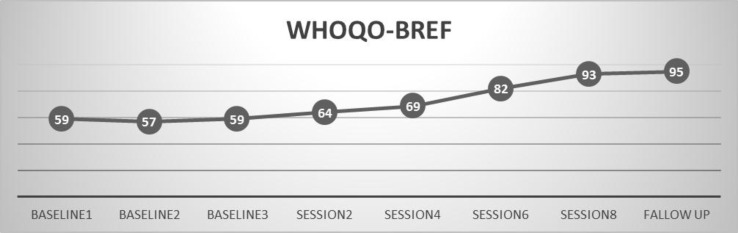
Quality of life in the three sessions before the intervention and eight session intervention for C mother with cerebral palsy child Based on WHOQOL-BREF, differences in 2, 4, 6, 8 sessions and follow up session were significant.

**Figure 4:**
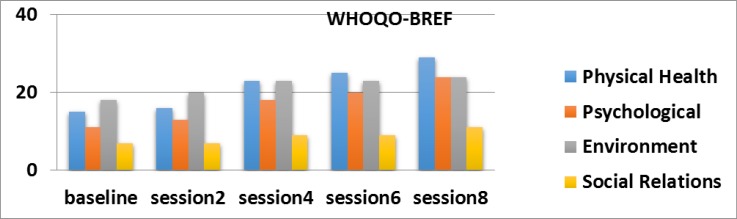
The sub - Domains of quality of life in the three sessions before the intervention and eight session intervention for A mother with cerebral palsy child Based on WHOQOL-BREF, differences in physical health and psychological field in 2, 4, 6, 8 sessions were significant.

**Figure 5:**
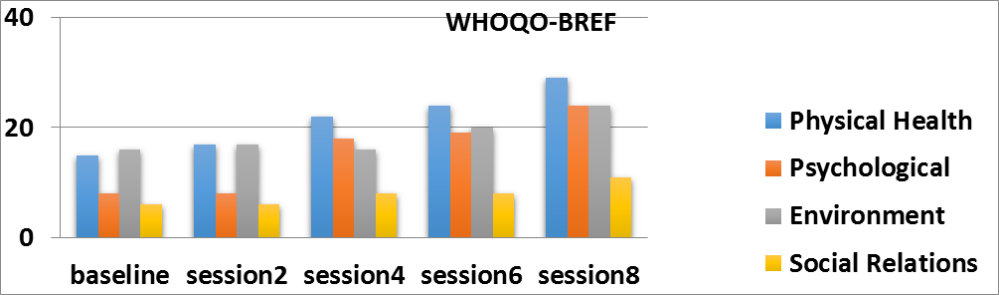
The sub - Domains of quality of life in the three sessions before the intervention and eight session intervention for B mother with cerebral palsy child Based on WHOQOL-BREF, differences in physical health and psychological and field in 2, 4, 6, 8 sessions were significant.

**Figure 6:**
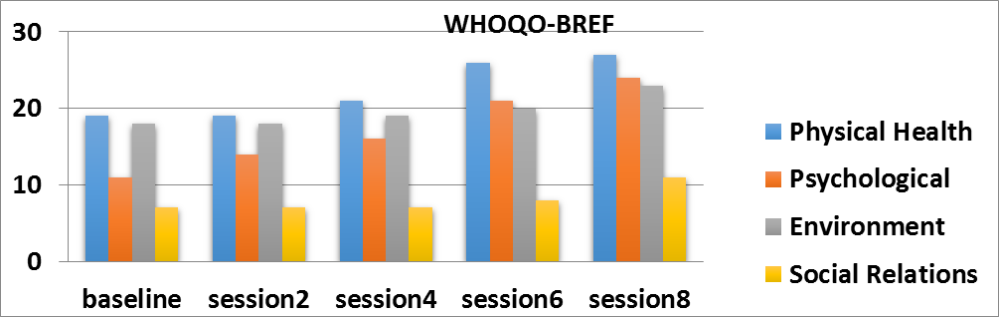
The sub - Domains of quality of life in the sessions before the intervention and eight session intervention for C mother with cerebral palsy child Based on WHOQOL-BREF, differences in physical health and psychological field in 2, 4, 6, 8 sessions were significant.

## Discussion

Cerebral palsy is a disorder that may seriously affect many aspects of the lives of individuals suffering from this disorder, and may also markedly reduce their health-related QOL. Hence, an intervention that contributes to decreasing the negative effects of CP is urgently needed. This study protocol describes a trial that will test MBCT as an intervention for caregivers of CP children, with the primary aim of reducing the negative effects of this disorder on the lives of the mothers of CP children. 

Overall, the results of this study indicated that MBCT was effective in helping the participants to deal with their anxiety, depression and stress, and it also helped them experience an improved quality of life before, during and after stressful circumstances; this is in line with the Baer’s review of Mindfulness-Based Interventions. In this study, the average effect Size was 0.71 for pre-post change across a range of physical and psychiatric disorders, and the effect size was 0.90 where depression was included as a dependent measure. The outcomes in the present study were comparable to the previous reports (12, 22, 30-32).

 The present study addressed the effect of MBCT on quality of life of mothers with CP children. This effect was observed as the quality of life of this group of mothers increased.

The MBCT program provides systematic training in mindfulness as a self-regulation strategy to reduce stress and manage emotion. This program is intended to foster greater awareness of what happens in each moment through the application of an attitude of acceptance. MBCT is designed to help people avoid habitual negative thoughts, emotions and behavioral patterns. Instead, increased awareness and acceptance are seen as allowing for new ways to respond and cope with different situations both in relation to oneself and the wider world. Mindfulness training has been linked to the changes in those areas of the brain which are responsible for affect regulation and stress impulses reactions; in turn, these changes influence body functions such as breathing, heart rate and immune function. Thus, as a result of MBCT trainings, the participants presumably acquired the skills to disengage themselves from ruminative thoughts and images that confer some protection against future stressful situations and subsequent stress, anxiety and depression (16, 27, 33). In line with the results of previous studies, our study supports the effectiveness of mindfulness-based cognitive therapy in enhancing the quality of life of mothers of children with cerebral palsy.

**Constraint**

A number of methodological limitations need to be considered; the most important of which, was lack of a control group, meaning that the benefits (and setbacks) observed in the participants cannot be conclusively assigned to the treatment.

## Conclusions

The present study is important in showing that mindfulness and self-control make significant independent contributions to psychological health quality of life for mothers of children with cerebral palsy. Finding ways to enhance quality of life would be a good direction for future research. Perhaps in this way we can to help this group of women despite the presence of the disorder in their lives, have a better quality of life and consequently, they provide better quality of life for children and the whole family.
